# The Lambda Variant in Argentina: Analyzing the Evolution and Spread of SARS-CoV-2 Lineage C.37

**DOI:** 10.3390/v15061382

**Published:** 2023-06-16

**Authors:** Mercedes Soledad Nabaes Jodar, Carolina Torres, Laura Mojsiejczuk, Dolores Acuña, Laura Elena Valinotto, Stephanie Goya, Monica Natale, Silvina Lusso, Sofia Alexay, Ariel Amadio, Matias Irazoqui, Franco Fernandez, Maria Elina Acevedo, Cristina Alvarez Lopez, Andres Angelletti, Paula Aulicino, Elisa Bolatti, Bettina Brusés, Marco Cacciahue, Ana Cavatorta, Agustina Cerri, Andres Cordero, Humberto Debat, Maria Jose Dus Santos, Maria Florencia Eberhardt, Regina Ercole, Carlos Espul, Marisa Farber, Fabián Fay, Ailen Fernandez, Florencia Ferrini, Laura Formichelli, Santiago Ceballos, Fernando Gallego, Adriana Giri, Maria Gismondi, Raul Maximiliano Acevedo, Ivan Gramundi, María Eugenia Ibañez, Guido Konig, Viviana Leiva, Melina Lorenzini Campos, Horacio Lucero, Nathalie Marquez, Melina Mazzeo, Alicia Susana Mistchenko, Luciana Montoto, Marianne Muñoz, Victoria Nadalich, Cristina Nardi, Belén Ortiz, Luis Pianciola, Carolina Pintos, Andrea Puebla, Carolina Rastellini, Alejandro Ezequiel Rojas, Javier Sfalcin, Ariel Suarez, Clara Theaux, Guillermo Thomas, Estefania Tittarelli, Rosana Toro, Vanina Villanova, Gretel Wenk, Cecilia Ziehm, Maria Carla Zimmermann, Sebastian Zunino, Proyecto PAIS, Mariana Viegas

**Affiliations:** 1 Laboratorio de Virologia, Hospital de Ninos Dr. Ricardo Gutierrez, Gallo 1330, Ciudad Autónoma de Buenos Aires 1425, Argentina; 2 Consejo Nacional de Investigaciones Cientificas y Tecnicas (CONICET), Godoy Cruz 2390, Ciudad Autónoma de Buenos Aires 2915, Argentina; 3 Instituto de Investigaciones En Bacteriologia y Virologia Molecular (IbaViM), Junín 956, Ciudad Autómoma de Buenos Aires 1113, Argentina; 4 Instituto de Investigación de La Cadena Lactea (IDICAL) INTA-CONICET, Ruta 34 Km 227, Rafaela 2300, Argentina; 5 Instituto de Patología Vegetal, Centro de Investigaciones Agropecuarias, Instituto Nacional de Tecnologia Agropecuaria (IPAVE-CIAP-INTA), Camino 60 Cuadras Km 5,5, Córdoba 5020, Argentina; 6 Laboratorio de Salud Pública, Facultad de Ciencias Exactas, Universidad Nacional de La Plata, Calle 1 y 47, La Plata 1900, Argentina; 7 Laboratorio de Biología Celular y Retrovirus, Hospital de Pediatría Prof. Juan P. Garrahan, Avenida Brasil 1175, Ciudad Autónoma de Buenos Aires 1260, Argentina; 8 Grupo Virología Humana, Instituto de Biología Molecular y Celular de Rosario (CONICET), Suipacha 590, Rosario 2000, Argentina; 9 Instituto de Medicina Regional, Universidad Nacional del Nordeste, Av. Las Heras 727, Resistencia 3500, Argentina; 10 Instituto de Biotecnología, Instituto de Agrobiotecnología y Biología Molecular (INTA-CONICET), De Los Reseros y N. Repetto s/No, Hurlingham 1686, Argentina; 11 Centro de Tecnología En Salud Pública, Facultad de Ciencias Bioquímicas y Farmacéuticas, Universidad Nacional de Rosario (UNR), Suipacha 531, Rosario 2000, Argentina; 12 Instituto de Virología e Innovaciones Tecnológicas (INTA-CONICET), De Los Reseros y N. Repetto s/No, Hurlingham 1686, Argentina; 13 Laboratorio de Diagnostico-UNIDAD COVID, Universidad Nacional de Hurlingham, Hurlingham 1686, Argentina; 14 Laboratorio de Virología, HIEAyC San Juan de Dios, Calles 27 y 70, La Plata 1900, Argentina; 15 Dirección de Epidemiologia y Red de Laboratorios Del Ministerio de Salud de La Provincia de Mendoza, Mendoza 5500, Argentina; 16 CIBIC Laboratorio, Pte. Roca 746, Rosario 2000, Argentina; 17 Laboratorio Central Ciudad de Neuquén, Ministerio de Salud, Gregorio Martínez 65, Neuquén 8300, Argentina; 18 Laboratorio de Medicina Genómica, Facultad de Medicina, Universidad Nacional Del Nordeste, Córdoba 1430, Argentina; 19 Cadic-Conicet, Universidad Nacional de Tierra del Fuego, Houssay 200, Ushuaia 9410, Argentina; 20 Hospital Regional Ushuaia, Av. 12 de Octubre y Maipú, Ushuaia 9410, Argentina; 21 Instituto de Botánica Del Nordeste-UNNE, Sargento Juan Bautista Cabral 2131, Corrientes 3400, Argentina; 22 Biología Molecular-Laboratorio Central, Hospital Alemán, Av. Pueyrredón 1640, Cuidad Autónoma de Buenos Aires 1118, Argentina; 23 Laboratorio de Salud Pública, Talcahuano 2194, Godoy Cruz 5501, Argentina; 24 Comisión Investigaciones Científicas de La Provincia de Buenos Aires, Camino General Belgrano y 526, La Plata 1900, Argentina; 25 Laboratorio de Biología Molecular Hospital Pedro de Elizalde, Avenida Manuel A Montes de Oca 1402, Cuidad Autónoma de Buenos Aires 1270, Argentina; 26 Instituto de Ciencias Polares, Ambiente y Recursos Naturales (ICPA) de La Universidad Nacional de Tierra Del Fuego (UNTDF), Houssay 200, Ushuaia 9410, Argentina; 27 Departamento de Biología y Genética Molecular, IACA Laboratorios, San Martín 68, Bahía Blanca 8000, Argentina; 28 Laboratorio de Biología Molecular Del Hospital General de Agudos, Carlos G. Durand, Diaz Vélez 5044, Cuidad Autónoma de Buenos Aires 1405, Argentina; 29 Laboratorio Mixto de Biotecnología Acuática, Av. Eduardo Carrasco y Cordiviola, Rosario 2000, Argentina; 30 Laboratorio de Virología Molecular, Hospital Blas L. Dubarry, Calle 12 825, Mercedes 6600, Argentina; 31 Consorcio Argentino de Genómica de SARS-CoV-2, Proyecto Argentino Interinstitucional de Genómica de SARS-CoV-2, Gallo 1330, Ciudad Autónoma de Buenos Aires 1425, Argentina

**Keywords:** SARS-CoV-2, Lambda, variants, evolution, South America, phylodynamic

## Abstract

The second wave of COVID-19 occurred in South America in early 2021 and was mainly driven by Gamma and Lambda variants. In this study, we aimed to describe the emergence and local genomic diversity of the SARS-CoV-2 Lambda variant in Argentina, from its initial entry into the country until its detection ceased. Molecular surveillance was conducted on 9356 samples from Argentina between October 2020 and April 2022, and sequencing, phylogenetic, and phylogeographic analyses were performed. Our findings revealed that the Lambda variant was first detected in Argentina in January 2021 and steadily increased in frequency until it peaked in April 2021, with continued detection throughout the year. Phylodynamic analyses showed that at least 18 introductions of the Lambda variant into the country occurred, with nine of them having evidence of onward local transmission. The spatial–-temporal reconstruction showed that Argentine clades were associated with Lambda sequences from Latin America and suggested an initial diversification in the Metropolitan Area of Buenos Aires before spreading to other regions in Argentina. Genetic analyses of genome sequences allowed us to describe the mutational patterns of the Argentine Lambda sequences and detect the emergence of rare mutations in an immunocompromised patient. Our study highlights the importance of genomic surveillance in identifying the introduction and geographical distribution of the SARS-CoV-2 Lambda variant, as well as in monitoring the emergence of mutations that could be involved in the evolutionary leaps that characterize variants of concern.

## 1. Introduction

Since the onset of the COVID-19 pandemic, genome sequencing efforts around the world have monitored the spread and evolution of the SARS-CoV-2 virus. By this means, the introduction, circulation, and establishment of the SARS-CoV-2 lineages in each country were studied as well as the identification of specific mutations that impact virulence, pathogenesis, host range, immune evasion, as well as the effectiveness of diagnostic tests, vaccines, and therapeutic treatments [1]. 

Although there is an unprecedented number of genomes available, there still exists a significant disparity in the distribution of genomic data, especially in regions such as South America [2]. Until 26 April 2023, 405,692 whole-genome sequences from South America had been generated and shared in the GISAID database [3], accounting for 0.21% of all reported positive cases of SARS-CoV-2 from the continent. In comparison, regions with higher coverage include Europe, with 7,700,433 genomes (2.70% coverage), and North America, with 5,458,362 genomes (4.12% coverage) (WHO Coronavirus (COVID-19) Dashboard https://covid19.who.int/table (accessed on 26 April 2023). Several studies have highlighted the importance of genomic surveillance by using genomic data to examine the evolution and associated spread of dominant variants in specific countries or regions, which may be a key factor in the global community’s ability to contain and control infectious disease threats [4]. 

The detection of emerging viral variants of SARS-CoV-2 with characteristics of concern for public health has had a significant impact on the development of the pandemic [5]. A common characteristic of these variants is the presence of mutations at the Spike (S) glycoprotein [6]; particularly, the N-terminal domain (NTD), receptor-binding domain (RBD), and the Furin cleavage site have been identified as hotspots that concentrate amino acid changes. Several coincident amino acid changes in the SARS-CoV-2 Spike protein have independently emerged in different viral lineage, evidencing that these changes could confer an adaptive advantage towards the virus infectivity and easier spread in the population [7]. 

In late 2020, the Argentine Inter-Institutional SARS-CoV-2 Genomic Consortium (PAIS Consortium) implemented a molecular surveillance strategy that focused on monitoring viral variants of concern (VOC) and variants of interest (VOI) in Argentina. This allowed for monitoring the emergence of new local variants and facilitated genomic and evolutionary analyses to study their origin and dispersion within the country [8]. During the first half of 2021, the second wave of COVID-19 in Argentina occurred in the context of an ongoing vaccination campaign that began in December 2020, prioritizing strategic personnel and high-risk groups such as the elderly. By mid-2021, 20% of the population had received at least one dose of the COVID-19 vaccine, and only 7% had completed the full vaccination schedule [9]. Also, throughout 2021, the Argentine government continued with the implementation of a comprehensive plan for preventive social distancing as a critical strategy to mitigate the spread of COVID-19. This plan included significant restrictions on the mobility of people, primarily limiting movement to short distances, with interprovincial travel being the maximum allowed. International travel was mainly restricted to commercial activities, with air travel from Buenos Aires City being the primary mode of transportation. Land travel with neighboring countries was tightly regulated and limited to commercial activities. The government implemented these measures to reduce the risk of imported cases and local outbreaks, as well as to limit the transmission of the virus within the country [10].

During the period spanning from October 2020 to April 2022, a comprehensive surveillance program was implemented, involving the analysis of a total of 9356 respiratory samples collected from diverse regions of Argentina [11,12]. It was determined that all SARS-CoV-2 variants of global epidemiological importance, except for the Beta variant, were present and circulating within the country at some point during the study period [8].

Noteworthy, since mid-February 2021, a very rapid growth in the frequency of genomes carrying the nonsynonymous mutations S_L452Q and S_F490S in the Spike gene was observed in samples from the Metropolitan Area of Buenos Aires (MABA), which has been the epicenter of the epidemic in Argentina [11]. The complete genome analysis showed that these samples belong to the lineage C.37, later designated as a global VOI, and assigned the WHO label “Lambda” [13]. This variant was detected in several countries worldwide, but it has spread particularly rapidly and with growing frequency in South America, mainly in Peru, Argentina, and Chile. Remarkably, while the Alpha variant was responsible for most COVID-19 cases around the world during the beginning of 2021 [4], Lambda and Gamma were the most frequently detected variants in Argentina [11].

Here, we describe the emergence, spread, predominance, and decline of the Lambda SARS-CoV-2 variant in Argentina, and the relationship with global Lambda isolates. This study provides insights into the understanding of the evolutionary dynamics of a SARS-CoV-2 viral variant in a geographic region during social distancing measures and ongoing vaccination strategies.

## 2. Materials and Methods

### 2.1. SARS-CoV-2 Sample Collection and Sequencing

Since the start of the pandemic, molecular surveillance of SARS-CoV-2 virus has been implemented in Argentina by the PAIS Consortium. Fifteen sequencing nodes throughout the country have sequenced and analyzed the SARS-CoV-2 virus using two strategies: next-generation sequencing (NGS) to obtain complete genomes and Sanger sequencing to obtain a fragment of the Spike protein gene spanning amino acids (aa) 428 to 750, as previously reported in Torres et al. [11]. These approaches together provide valuable epidemiological information, allowing lineage distribution and virus genetic diversity to be characterized in near-real time. 

During the second wave of COVID-19, an intensive sampling strategy for sequencing was implemented, which involved randomly selecting a 2.5–10% fraction of the total positive cases detected weekly in different healthcare centers. This approach enabled the detection of emerging variants and the tracking of their frequency in various regions of the country. Regular sampling was conducted at sentinel laboratories, in addition to sporadic sampling in some locations. These samples were selected for sequencing after positive tests for SARS-CoV-2 were reported, considering both the Ct value (<30) and the availability of epidemiological metadata (date of collection and the place of residence of the patients). It is worth noting that all samples come from individuals who acquired the infection in the community, since those with a travel history abroad or related to travelers were excluded from the analyzed cohort. Depending on the sequencing node, libraries preparation and sequencing were performed either on Illumina and/or Oxford Nanopore Platforms. The preparation of SARS-CoV-2 genomic libraries was performed using two different strategies: (1) the Quick protocol [14] with Illumina platform and (2) the Oxford Nanopore sequencing using the ARTIC Network or Midnight primer scheme [15]. The libraries were sequenced on the Illumina MiSeq or NextSeq instruments (Illumina, San Diego, CA, USA) and in R9 flow cells on a MinIon device (Oxford Nanopore Technologies, Oxford, UK). 

The reads obtained from sequencing on the Illumina platform were initially filtered by quality, both at the base level and the read level. Subsequently, PCR duplicates, spurious primer sequences, and reads potentially contaminated from other organisms, particularly human contamination (using the DeconSeq program [16]), were removed using BBMap [17]. Finally, the quality of the resulting reads was assessed using the FastQC program [18]. The resulting reads were aligned to the SARS-CoV-2 reference genome (ID EPI_ISL_402124, hCoV-19/Wuhan/WIV04/2019) using the BWA-MEM software [19]. Finally, the consensus sequence for each sample was generated in FASTA format by the pile-up command of the samtools software and the consensus command of bcftools [20].

After sequencing on Oxford Nanopore Technologies (ONT) sequencing platforms, the raw sequencing data (files in FAST5 format) were converted into DNA sequences, and the index sequences were trimmed using the Guppy program [21]. The workflow known as “ARTIC SARS-CoV-2”, developed on the Oxford Nanopore EPI2ME platform [22], was used to obtain the complete viral genome as a FASTA-formatted sequence. In this protocol, DNA sequences in FASTQ format were filtered based on sequence length and quality and then aligned to the reference SARS-CoV-2 genome using minimap2. A specific bed file from the primer scheme was used to identify regions of the mapped sequences corresponding to synthetic sequences (primers), and these regions were trimmed to ensure that the sequences were entirely of biological origin. The retained sequences were used to generate a consensus sequence, which was further polished using Medaka [23].

A total of 9356 sequences were obtained by the PAIS Consortium, with 3531 obtained through NGS (complete genomes) and 5825 obtained through Sanger sequencing (fragment of the Spike protein gene).

### 2.2. SARS-CoV-2 Genomic Datasets

**Figure 1:**
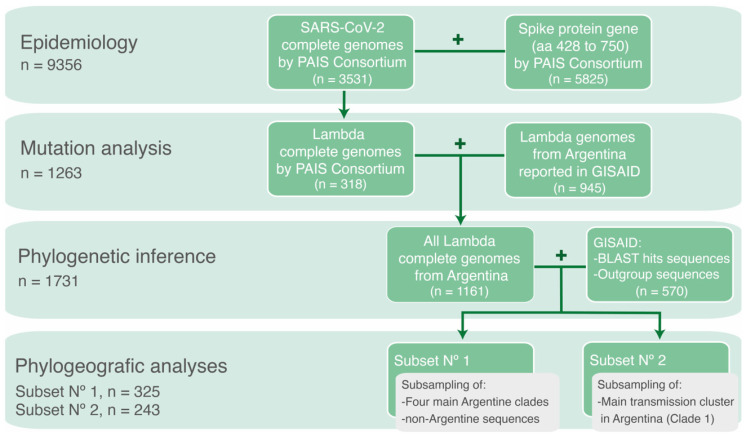
Schematic representation of the datasets used according to the specific objective of each analysis.

Different datasets were generated depending on the analyses to be performed. A schematic representation of the datasets is shown in Figure 1.

To analyze the genomic epidemiology of SARS-CoV-2 variants in Argentina during the circulation period of the Lambda variant, complete- and partial-genome sequences obtained by the PAIS Consortium between epidemiological week (EW)-44/2020 and EW-17/2022 were used. A total of 9356 sequences were analyzed, with 3531 obtained through NGS (complete genomes) and 5825 obtained through Sanger sequencing (fragment of the Spike protein gene). 

To analyze the mutational patterns within Lambda whole-genome sequences, all complete genomes of the Lambda variant obtained by the PAIS Consortium (*n* = 318) were combined with SARS-CoV-2 genomes reported by other groups in the GISAID database [3]. The search criteria were set to include genomes with a collection date between EW-44/2020 and EW-17/2022, located in Argentina, and classified as the Lambda variant (lineage C.37) using the Pangolin COVID-19 Lineage Assigner (accessed on 30 April 2022) [24]. This dataset comprised all Lambda genome sequences from Argentina throughout the studied period (*n* = 1263).

To study the introductions and diversification of Lambda in Argentina, a phylogenetic analysis was conducted with the 1263 genome sequences of the Lambda variant from Argentina, along with their most similar sequences (the best ten hits for each sequence) from a BLAST analysis conducted against the GISAID database (accessed on 30 April 2022), plus reference sequences from other lineages as outgroup. Sequences <29 Kb were excluded, except for isolates with 27–29 Kb in length that were kept due to the scarce genomic data available from some regions. The final dataset included 1731 sequences, with 1688 Lambda sequences.

Finally, to reconstruct the spatiotemporal distribution of the Lambda variant in Argentina, two subsets were generated by subsampling sequences previously used in the phylogenetic analysis. This approach was carried out to optimize the computational process required for the Markov chain runs and ensure convergence of the analysis. The first analysis comprised a subset of the sequences (*n* = 325) used in the previous phylogenetic analysis of the four main phylogenetic clades of Lambda in Argentina. The selection process considered the geographic distribution and collection dates of the sequences. As a result, redundant sequences were removed if they originated from the same location, had very similar collection dates, and clustered together within a phylogenetic clade. This subset was used to estimate the time and location of the most recent common ancestors, rates of evolution and demographic reconstruction. The geographic distribution of the Argentine sequences included in this analysis is described in the phylogeographic analysis (Section 3.4). The second analysis consisted in a different subset of sequences used in the phylogenetic analysis, focusing on the main transmission cluster in Argentina (*n* = 243). This subset was also chosen with consideration for geographic representation and collection dates to conduct a Bayesian phylogeographic diffusion analysis in discrete space.

We gratefully acknowledge the authors from the originating laboratories responsible for obtaining the specimens and the submitting laboratories where genetic sequence data were generated and shared via the GISAID Initiative, on which part of this research is based. All genome sequences and associated metadata in these datasets are published in GISAID’s EpiCoV database. To view the contributors of each individual sequence with details such as accession number, Virus name, Collection date, Originating Lab and Submitting Lab and the list of Authors, visit 10.55876/gis8.230503du.

### 2.3. Phylogenetic Inference

Sequences were aligned using MAFFT v7.475 [25] with the default parameters and manually edited to exclude the 5′ and 3′ ends (first 54 nt and the last 67 nt, in reference to isolate hCoV-19/Wuhan/WIV04/2019, EPI_ISL_402124). The best-fitted evolutionary model was determined using ModelFinder [26] according to the Bayesian Information Criterion (BIC). Maximum-likelihood phylogenetic reconstruction was performed with the IQ-TREE COVID-19 release 2.2.0 [27] and clustering confidence was evaluated using the Ultrafast Bootstrap approximation method (UFBoot, 10,000 replicates) [28] and Shimodaira–Hasegawa approximate likelihood ratio test (SH-aLRT, 1000 replicates) [29].

To verify the lineage assignment made by Pangolin COVID-19 Lineage Assigner, maximum-likelihood phylogenetic trees were performed (data not shown), including all Argentine sequences and reference sequences for several lineages and sublineages obtained from the PANGO designation list v1.9 [30]).

### 2.4. Phylodynamic Analysis

A Bayesian coalescent approach was used to co-estimate the temporal and spatial history of the SARS-CoV-2 C.37 lineage in Argentina. In addition, a discrete phylogeographic analysis was performed and used to visualize the spatiotemporal spread of the main transmission cluster in Argentina. 

The temporal signal of the datasets was examined by root-to-tip regression using Tempest v1.5.1 [31] software. Sequences whose genetic divergence and sampling date were incongruent (according to visual inspection) with the general pattern of the datasets were discarded, as they could have had sequencing errors, contamination, or misassigned collection date.

The analyses were carried out using an appropriate substitution model according to the BIC estimated with ModelFinder in IQ-TREE v2.1 [26,27]. The uncorrelated lognormal (UCLN) molecular clock model [32], and the Bayesian Skyline coalescent model [33] implemented in the BEAST v1.10.4 software package [34], were used. A discrete phylogeographic model with an asymmetric substitution matrix over the sampling locations was set, with all transitions equally probable as a prior. 

Three independent Markov chain Monte Carlo (MCMC) chains were run for each dataset. Results were examined with Tracer v1.7.1 [35] to evaluate the convergence of parameters (effective sample size ≥200, acceptable mixing without tendencies in traces, with a burn-in of 10%) and concatenated with LogCombiner [34]. Uncertainty in parameter estimate was evaluated in the 95% highest posterior density (HPD95%) interval. The maximum clade credibility tree (MCCT) was summarized using Tree Annotator v1.10.5 [34], visualized with FigTree v1.4.4 [36], and analyzed further in the SPREAD3 program [37].

### 2.5. Mutation Analysis

To study the emergence of mutations in Argentina, we traced the profiles of nonsynonymous mutations and compared their frequency in the studied population. The mutational profile of each sequence from lineage C.37 was investigated using the CoVsurver tool available at the GISAID EpiCoV platform [3] to identify nonsynonymous changes in comparison with the Wuhan-Hu-4 reference sequence (GISAID: EPI_ISL_402124). All frequencies were calculated based on the coverage of each position in the genome sequence, and those positions with less than 50% coverage were excluded from the analysis.

### 2.6. Statistical Analysis

The frequencies of detection of VOI, VOC, or mutations and the 95% CIs were estimated with the Wilson/Brown method, implemented in the GraphPad Prism v.9.2 program (San Diego, CA, USA).

### 2.7. Data Visualization 

The plots were generated using graphical visualization tools at covdb.stanford.edu [38] (Philip L. Tzou et al., 2022) and/or Microsoft Excel v16.47.1 and edited in Adobe Illustrator 23.1.1.

## 3. Results

### 3.1. The Molecular Epidemiology of Lineage C.37 in Argentina

**Figure 2:**
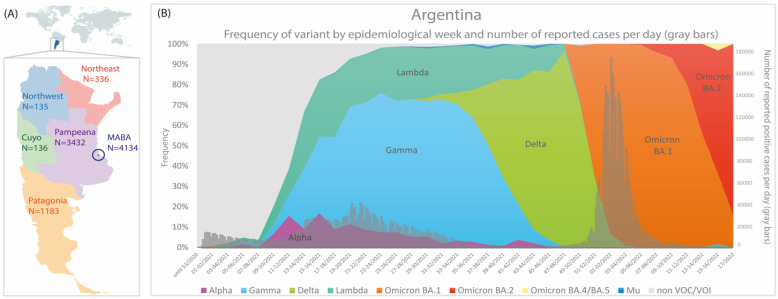
(**A**) Number of partial/complete genomes by Argentine regions. (**B**) Frequency of SARS-CoV-2 variants and number of reported cases per day by epidemiological week (*n* = 9356); only cases that did not present a history of travel or close contact. The colors in (**B**) represent the cumulative abundance of each SARS-CoV-2 variant per epidemiological week.

From October 2020 to April 2022, 9356 respiratory samples positive for SARS-CoV-2 from Argentina were subjected to sequencing, out of which 3531 were obtained through NGS and the remainder were obtained through Sanger sequencing. In this study, we analyzed genomic sequences obtained from the six regions of the country [38], but with a heterogeneous distribution. A high number of genomes from the MABA and Pampeana regions were sequenced, while in regions such as Cuyo, Northeast, and Northwest, a lower number of sequences were obtained due to their lower population and, as a consequence, a lower total number of cases (Figure 2A) [39].

Of note, 82.04% of the samples corresponded to a VOC/VOI: 26.96% Gamma, 21.37% Omicron, 16.10% Delta, 13.24% Lambda, 4.19% Alpha, and 0.18% Mu (Figure 2). In previous works, we have reported the emergence and evolution of SARS-CoV-2 lineages during the first and third waves of COVID-19 in Argentina [11,12,40]. In this study, we have focused on the analysis of the Lambda variant during the second wave of COVID-19 in Argentina that took place during the first half of 2021.

The Lambda variant was first detected in Argentina on 30 January 2021, through the detection of its characteristic mutations in the Sanger-sequenced region of Spike protein gene (S_L452Q and S_F490S). Since then, it was detected in 1239 out of 9356 genome sequences from Argentina with a frequency that increased steadily since February (EW-5/2021) and reached a value of 31.1% (EW-17/2021, end of April) (Figure 2B and Table S1). However, the impact of the Lambda variant was heterogenous in each region of the country, and the highest frequencies were found in the MABA (44.7%, SE 17-18/2021) and Pampeana regions (27.4%, SE 33-34/2021).

By the end of October 2021, the Lambda frequency decreased to values below 10% in accordance with the end of the second wave of COVID-19 and the introduction of the Delta variant in Argentina. Since then, Lambda was detected only in sporadic cases until April 2022. Notably, the last detected case occurred during the third wave of COVID-19, when the Omicron variant had a prevalence above 99% since EW 03-04/2022. The clinical–epidemiological investigation revealed that this single case was an immunocompromised individual with an advanced HIV infection. Indeed, a particular set of mutations was found in the genome sequence, which indicates a highly divergent Lambda variant that has not yet been reported in databases. 

### 3.2. Phylogenetic Analysis

Argentine sequences were analyzed along with the most similar sequences in the GISAID database to infer common transmission chains and establish the presence of circulating viruses. The Argentine sequences included in this analysis were distributed across regions as follows: 35% Pampeana, 26% Northwest, 15% MABA, 11% Patagonia, 9% Northeast, and 4% Cuyo.

**Figure 3:**
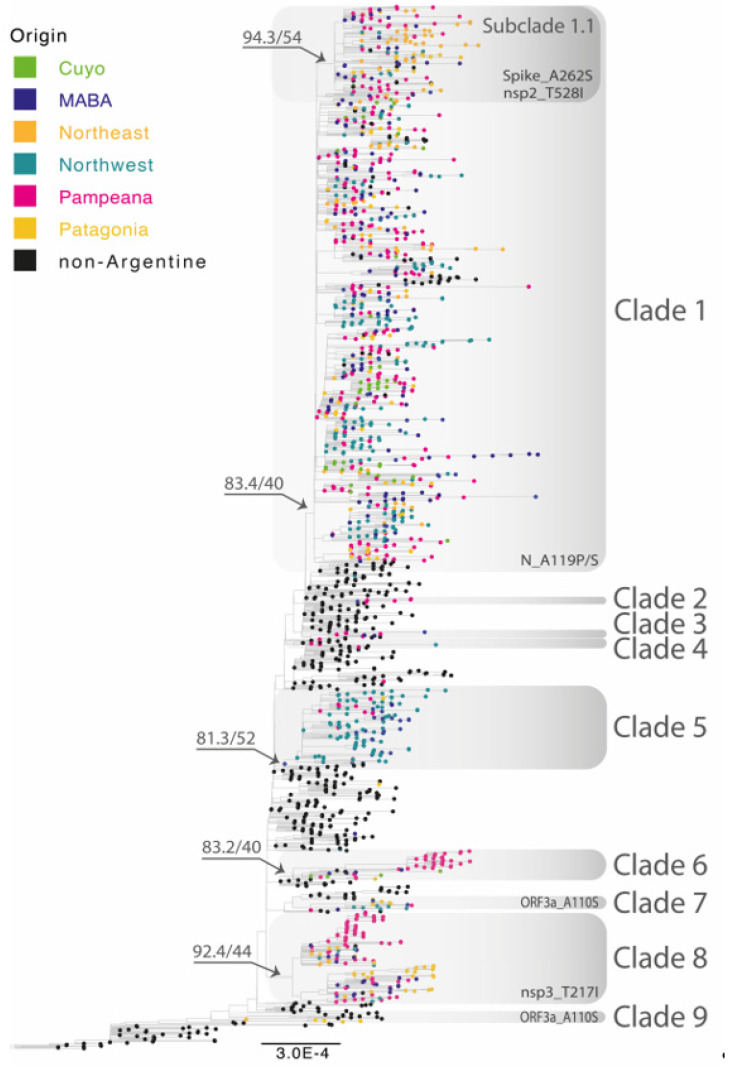
Maximum-likelihood phylogenetic tree of SARS-CoV-2 whole-genome sequences of Lambda (lineage C.37). Tips are colored by region and clades with Argentine genomes are highlighted by grey rectangles. Nonsynonymous mutations associated with one or more of the Argentine clades are shown at the bottom of the rectangles. B.1.1.1 sequences were used as the outgroup. The SH-like/UFB values for the relevant groups are indicated for some groups. UFB: ultrafast bootstrap. The scale indicates the number of substitutions per site.

The introduction and diversification study performed on the Argentine Lambda genomes revealed a minimum of 18 independent introduction events, 9 of them with evidence of onward transmission. Most Lambda sequences from Argentina clustered into four main clades, labeled as 1, 5, 6, and 8, where samples from all Argentine regions were found (Figure 3). Clades 5, 6, and 8 were associated with specific regions: Clade 5 circulated mainly in the Northwest region, Clade 6 mostly in the Pampeana region, and Clade 8 between the Patagonia and Pampeana regions. Regarding Clade 1, the largest one, the 861 Argentine sequences were split into several subclades, with few sequences collected from other countries (mostly the USA, Chile, Spain, and Mexico). Although the basal relationship between subclades could not be resolved with confidence, highly supported local groups with viral diversification to different cities could be identified (complete full-detailed phylogenetic tree is in the Supplementary Materials). 

In addition to these major clades, we also identified a number of smaller clades (2, 3, 4, 7, and 9), as well as 7 singletons which were randomly interspersed with Lambda sequences mostly from Peru, Chile, and the United States. 

Notably, Clade 4 contained the latest Lambda sequence reported in Argentina in April 2022, PAIS-G1123 (EPI_ISL_13466784). This clade included 5 other sequences from Argentina, Chile, and the United States, all reported between February and July 2021. As expected, the branch containing PAIS-G1123 shows a large number of substitutions per site compared to the rest of the group.

### 3.3. Mutational Patterns in Argentine SARS-CoV-2 Whole-Genome Sequences

**Figure 4:**
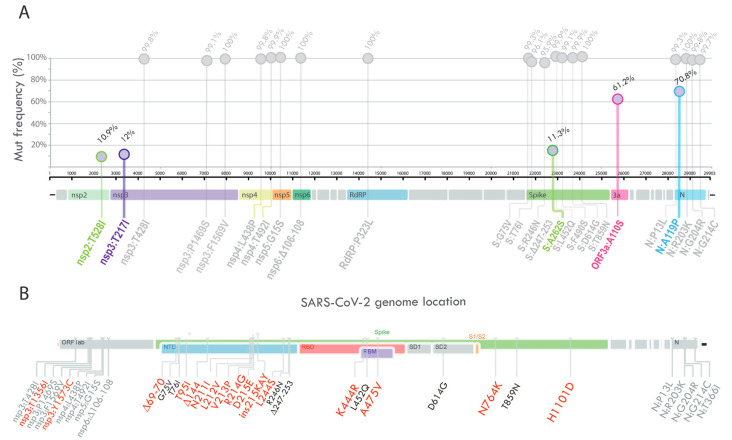
(**A**) Lollipop plot summarizing the frequency of SARS-CoV-2 nonsynonymous mutations and deletions with >10% prevalence. The bubbles’ y-coordinates indicate mutation frequencies, which are also shown above the bubbles. The five amino acid substitutions with frequency values between 10% and 90% are shown in colors. (**B**) Schematic summary of the changes found in the PAIS-G1123 sequence. Text in grey indicates the constellation of mutations characteristic of the Lambda variant, and red text indicates amino acid positions with changes that are unique to the PAIS-G1123 sequence compared to the most-related Argentine genome sequences of the Lambda variant.

Compared to the reference sequence hCoV-19/Wuhan/WIV04/2019 (GISAID accession number EPI_ISL_402124), the 1263 genome sequences displayed a total of 1631 amino acid changes in different viral genes (912 in ORF1a/1b, 268 in S, 121 in N, 114 in ORF3a, 94 in ORF7a, 47 in ORF8, 28 in M, 18 in ORF6, 17 in ORF7b, and 12 in E). In the Argentine sequences, all the C.37 lineage defining amino acid changes [41], shown in grey in Figure 4A, were found at a frequency higher than 95.9%. 

Five amino acid substitutions, with frequency values between 10 and 90% (shown with colors in Figure 4A), were further analyzed to determine if they could be assigned to one or more of the Argentine clades identified by phylogenetic inference (in Figure 3, the nonsynonymous mutations related to each clade are shown). While the amino acid substitution ORF3a_A110S was observed in several clades with Argentine sequences (clades 1, 7, and 9), the remaining mutations were found to be associated with specific Argentine clades. For instance, the amino acid substitution nsp3_T217I was only found in Clade 8, which includes genome sequences mainly from two regions, Pampeana and Patagonia. In relation to Clade 1, an amino acid replacement was found throughout the clade at position N_A119, of which more than 70% corresponded to the N_A119P substitution. In addition, the Spike_A262S and nsp2_T528I mutations were found as signatures of Subclade 1.1.

The analysis of the complete SARS-CoV-2 genome PAIS-G1123 (EPI_ISL_13466784) from the patient detected in April 2022 showed a total of 30 nucleotide substitutions, 4 deletions, and 1 insertion compared to the reference sequence hCoV-19/Wuhan/WIV04/2019 (EPI_ISL_402124). Of these, 19 mutations correspond to the constellation of mutations characteristic of the Lambda variant. Interestingly, this case occurred at a moment of no circulation of Lambda in our country, suggesting a prolonged infection time that started months before their detection. Remarkably, compared to the most related genome sequences of the Lambda variant, this sequence displayed 13 nonsynonymous mutations, 2 deletions, and 1 insertion distributed in 2 genes: ORF1ab (replicase polyprotein) and Spike glycoprotein gene (Figure 4B). Of the 13 changes in the Spike protein, 2 were in the receptor-binding domain, 10 were in the N-terminal domain, and 2 were in the intergenic region (shown in red text, Figure 4B).

### 3.4. Phylogeographic Analyses

A discrete phylogeographic analysis was performed on a subset of Lambda sequences (*n* = 325), including the four main Argentine clades that showed local transmission (clades 1, 5, 6, and 8). The Argentine sequences included in this analysis were distributed across regions as follows: 26% Pampeana, 24% Northwest, 23% MABA, 11% Patagonia, 10% Northeast, and 6% Cuyo.

The Lambda variant displayed a rate of evolution of 3.88×10^−4^ substitutions per site per year (s/s/y) (HPD95% = 3.54 × 10^−4^ − 4.22 × 10^−4^), with an estimated date for its most recent common ancestor on 13 November 2020 (HPD95% = 15 October to 9 December 2020), placed in Peru (posterior probability (pp) 1.0).

**Figure 5:**
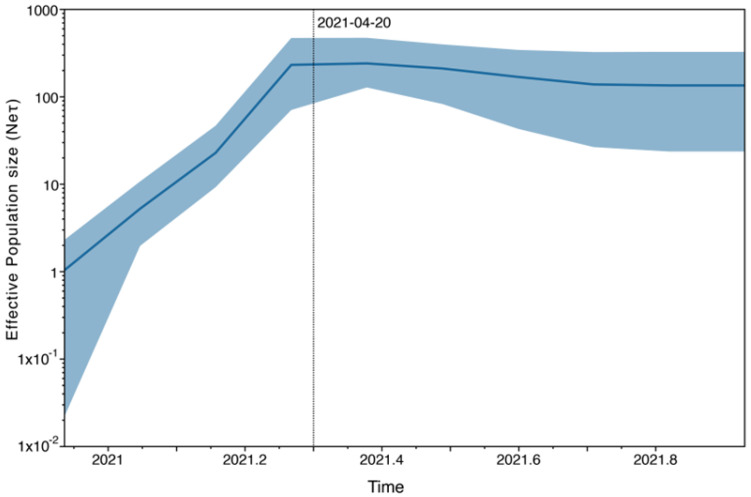
Coalescent Bayesian Skyline analysis. The black line is the mean estimate of the estimated effective population size. The two blue lines are the upper and lower bounds of the 95% HPD interval. The *x*-axis is the time in years, and the *y*-axis is on a log scale.

The demographic reconstruction of the Lambda isolates, represented by the Bayesian skyline plot, reveals a notable increase in the effective number of infections during the period spanning from December 2020 to mid-April 2021, when it reached a plateau continuing until the last sampling time (Figure 5).

**Figure 6:**
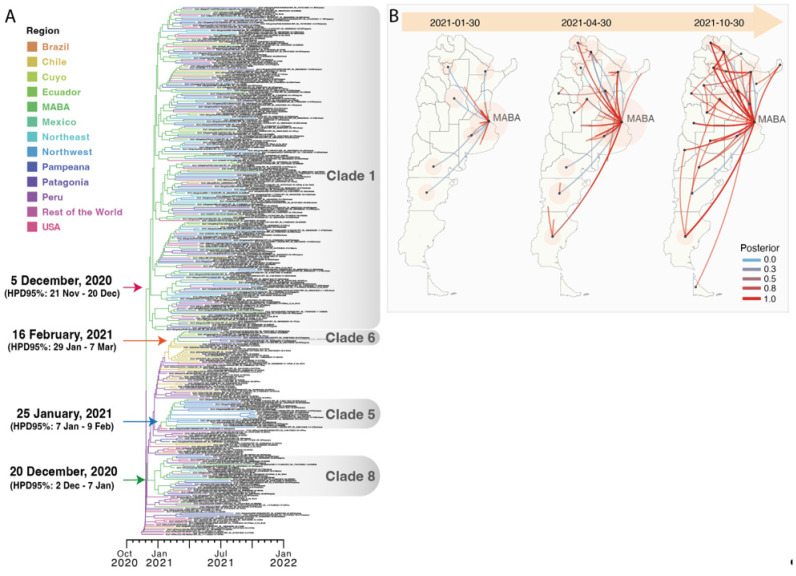
(**A**) The Bayesian discrete phylogeographic analysis. Maximum clade credibility tree for the Lambda variant. The branches’ colors represent the MRCA’s location (described in the legend). The time scale in years is detailed at the bottom. (**B**) Different stages of phylogeographic history of Clade 1 under a discrete diffusion model. The lines colors represent the posterior probability support for each transition rate between locations calculated by the BEAST program and summarized by the SPREAD3 program (described in the legend). The size of the polygons around a sampling location is proportional to the number of lineages that maintain that location.

To explore the time/date of the introduction of the viruses that gave rise to the four main clades that circulated in Argentina, we estimated the time of the most recent common ancestor (TMRCA) and its location for each clade (Figure 6A).

The TMRCA of Clade 1 was estimated on 5 December 2020 and was in MABA (pp, posterior probability, 0.84). To evaluate the regional distribution of this clade, which was the main clade of Lambda in Argentina, a second discrete phylogeographic analysis was conducted. The reconstruction of the diffusion history of Clade 1 is shown in Figure 6B. The result suggested an early spread of Lambda from MABA to other provinces that persisted throughout 2021, with a more limited onward spread among provinces since early May. 

The TMRCA of Clade 5 was estimated to be on 26 January 2021, and was located in Peru (pp 0.84). An Argentine sequence with a travel history to Peru was placed basally to this clade on the tree, so this sequence is likely to share a common ancestor with the probable source of introduction. Viruses within this transmission cluster were diversified predominantly in the Northwest region.

The TMRCA of Clade 6 was estimated to be on 16 February 2021, and was located in Chile (pp 0.92). Viruses in this cluster were distributed among all Argentine regions but were particularly widespread in the Pampeana region, where transmission was persistent until December 2021.

The TMRCA of Clade 8 was estimated to be on 20 December 2020. The location of the MRCA (Most Recent Common Ancestor) was estimated to be in MABA (pp 0.99), from where viruses within this cluster were mainly distributed between two Argentine regions (Pampeana and Patagonia), resulting in two highly supported chains of transmission. 

## 4. Discussion

Since the C.37 lineage of SARS-CoV-2 was initially reported in Peru in December 2020 [42], considerable research has been conducted, primarily in Peru, to elucidate the dispersion and evolutionary dynamics of the Lambda variant during the early stages of viral circulation [43,44]. However, the transmission and evolutionary dynamics of this variant in other affected regions remain incompletely characterized. To address this knowledge gap, our study aimed to comprehensively characterize the emergence and spread of the SARS-CoV-2 Lambda variant throughout Argentina, from its initial entry into the country until its detection ceased. Our investigation provides insights into the genetic and epidemiological features of the Lambda variant, contributing to a more comprehensive understanding of one of the SARS-CoV-2 variants of interest that had a major impact on South America, a region that has been less studied during the COVID-19 pandemic in comparison with higher-income countries.

Our analysis of 9356 SARS-CoV-2-positive respiratory samples revealed a consistent increase in the frequency of the Lambda variant from February 2021 (EW-5/2021) through the end of April (EW-17/2021) in Argentina. These findings were consistent with the earlier emergence of the Lambda variant in Peru, where the first case was detected in November 2020, and its frequency increased until April 2021 [45]. Lambda became the predominant variant in the Coastal and Andean regions of Peru during the first half of 2021, surpassing Gamma in almost the entire country for several months, likely because the Lambda variant held off Gamma expansion initially due to a “founder effect” (having been detected one month earlier) [44]. In contrast, in Brazil, a low level of Lambda VOI circulation was found, and Gamma was the dominant variant during early 2021 [46]. Argentina was the region where these two scenarios converged, as Lambda and Gamma cocirculated during the first half of 2021, with Gamma having a higher detection frequency. Given that Argentina shares large borders with other Latin-American countries, commercial contact with neighboring countries via terrestrial transportation may have led to the introduction and dissemination of both variants in the country. This phenomenon was also observed at the borders of the Amazon region of Peru with Brazil, where the circulation of the Gamma variant predominated in contrast to the rest of Peru [44]. Further information on the circulation of these variants in neighboring countries of Argentina, such as Bolivia or Paraguay, may confirm this hypothesis. In contrast, Alpha, which was the prevalent variant in Europe, had a minimum impact in Argentina, possibly because the closure of air borders possibly mitigated the epidemiological impact [47,48].

The demographic reconstruction of the Lambda variant showed an increase in the effective number of infections that corresponded with the increase in Lambda frequency in our dataset and with the reported cases in the National Surveillance System during the second wave of COVID-19 in Argentina [39]. In early April 2021, the national government decided to increase movement restrictions within the country and to cancel all flights to Latin-American countries due to the epidemiological situation in South America [49]. This may be reflected in the demographic reconstruction of the Lambda variant, where the plateau observed at the end of April could indicate a stabilization in the diversification of the Lambda variant due to increased social distancing and restrictions on foreign travel entry.

The persistence of the Lambda variant’s detection continued until October 2021, subsequently resulting in a decrease in its frequency to less than 10%. This decline coincided with the end of Argentina’s second wave of COVID-19 and the emergence of the Delta variant. The spread of Delta throughout Argentina started in August 2021, later than in other countries [50,51,52], and, like neighboring countries, the emergence of Delta did not lead to an increase in the incidence of COVID-19 cases and deaths (Figure 2). This could be a consequence of the strict limitations imposed on air travel, which also enabled higher vaccination rates before the entry and dominance of Delta in the country [53].

Our phylogenetic analyses of all reported Lambda sequences from Argentina indicated that there were at least 18 introductions into the country. The analyses showed the national spread of Lambda, as indicated by several large clades of closely related SARS-CoV-2 lineages, which were defined by mixtures of samples from patients living in different regions of Argentina. Also, smaller clades clustering within specific regions were observed, providing evidence of local transmission chains of the virus. These findings were consistent with interprovincial community transmission within the same region, such as that observed in the Northwest region (Clade 5) and Pampeana region (Clade 6), as movement to other regions was increasingly restricted since the emergence of Lambda in the country [49]. Clades with highly moderate support, including sequences from Argentina, were observed. However, the basal relationship between the clades could not be confidently resolved.

The phylogeographic analyses showed that the four main Argentine clades had an estimated date for their most recent common ancestors between December 2020 and February 2021; this finding is consistent with the detection of the first sample in the country for each clade occurring two months later. In clades 5, 6, and 8, the estimated location was in the neighboring countries, Peru and Chile. Interestingly, sequences from MABA were observed in all clades, often in basal positions, and given that the Lambda variant was first detected in MABA, this observation could suggest the significant role played by the pandemic epicenter of Argentina as the point of entry and subsequent transmission of the virus across the country. Moreover, in Clade 1, our phylodynamic analysis suggested an initial diversification in MABA, which subsequently spread to other regions within Argentina, leading to widespread transmission of the Lambda variant within the country, as shown in the spatiotemporal reconstruction of the dispersal history of Lambda in Figure 6B.

The Lambda variant is characterized by multiple lineage-specific deletions and amino acid substitutions in its viral genome, as shown in Figure 4A [41]. Specifically, the spike protein of the Lambda variant has a unique pattern of eight mutations (G75V, T76I, R246N, Δ247-253, L452Q, F490S, D614G, and T859N). Interestingly, the amino acid substitution in the L452 position had also been reported in the Delta and Omicron variants [54,55], and it had been demonstrated that the L452Q mutation conferred increased viral infectivity and resistance to vaccine-induced antiviral humoral immunity [56]. While L452Q is almost exclusive to C.37, L452R is present in Delta and former variants of interest Epsilon (B.1.427/B.1.429) and Kappa (B.1.617.1) and is associated with increased affinity for the human ACE2 receptor [57]. Furthermore, the F490S mutation had been associated with escape from convalescent sera [58]. The 247-253 deletion in the NTD of the Lambda Spike is located in an antigenic site, suggesting that this deletion might have contributed to immune escape by shortening or fully deleting neutralizing epitopes or exploiting increased glycosylation [59,60,61,62]. Lambda also displays the nsp6 106–108 deletion, found in VOCs alpha, beta, and gamma [63]. Given that the mutations present in Lambda and their genomic location are shared in many cases with the mutations found in the designated variants of concern, a greater epidemiological impact of the Lambda variant could have been observed if molecular surveillance in Latin America had been comparable to that of higher-income countries. Additionally, the government’s implementation of containment measures to prevent the spread of COVID-19 may have contributed to the decrease in Lambda transmission to other continents.

Some of the mutations found in Argentine sequences were assigned to one or more of the Argentine clades identified by phylogenetic inference, such as Clade 1 (N_A119P, Spike_A262S, and nsp2_T528I), Clade 8 (nsp3_T217I), and clades 1, 7, and 9 (ORF3a_A110S). In accordance with our results, it has been reported that the genes encoding the nonstructural protein NSP3 and the structural proteins S and N exhibit the highest number of mutations in the Lambda sequences isolated from Peru, and that the proteins with some sites under positive natural selection were ORF3a, ORF8, and S [45].

The Lambda sequence, PAIS-G1123 (EPI_ISL_13466784), was detected in April 2022, several months after the last Lambda sequence was reported in Argentina and exhibited 14 nonsynonymous mutations, two deletions, and one insertion in comparison to the most closely related genome sequences of the Lambda variant detected in Argentina. The most remarkable characteristic of this genome is the acquisition of multiple mutations within the NTD of the S glycoprotein, including deletions at positions 69–70 and 144. These deletions have been identified in the Omicron and Alpha variants of concern and have been associated with an increase in infectivity as a result of enhanced incorporation of cleaved spike into virions and evasion of antibody response [64,65]. Given that the sample belonged to an immunocompromised patient, it is likely that the changes in the amino acid composition of the viral Spike protein were a result of an ongoing interaction between the persistent virus and the patient’s adaptive immune system, as has been documented in other comparable clinical cases [66,67]. The emergence of rare mutations in chronically infected immunocompromised hosts, implying a radical difference in selective pressure compared to common cases, could be involved in the evolutionary leaps that characterize variants of concern. Therefore, understanding the evolution of SARS-CoV-2 and the emergence of new variants through studies is critical in developing effective prevention and control strategies to minimize the impact of the virus on public health.

This study has contributed to the understanding of the evolutionary trends exhibited by SARS-CoV-2 and emphasizes the role of genomic surveillance in identifying the emergence and geographical distribution of the SARS-CoV-2 Lambda variant. Furthermore, it underscores the significance of intercountry interactions, thereby highlighting the importance of implementing monitoring measures, not only at the local but also at border level. The ecological competition between two viral variants, Lambda and Gamma, within a vaccinated population raises intriguing questions, as the mutations within these variants could potentially play a critical role in determining their competitive advantage. Moreover, the study investigates the emergence of intravariant mutations, along with their occurrence in immunocompromised patients. These patients serve as reservoirs for viral diversity, amplifying the significance of mutations observed within their cases. This emphasizes the imperative need for comprehensive monitoring and a thorough understanding of viral dynamics in both the general and immunocompromised populations.

We believe that increasing data sharing and research in South America is crucial to enhance our comprehension of the COVID-19 pandemic in this region. Furthermore, revealing the virological characteristics of mutations in VOCs and VOIs can establish a baseline, which is necessary to evaluate the risk of newly emerging SARS-CoV-2 variants in the future, as well as their impact on therapeutics or vaccination effectiveness.

## Data Availability

All genome sequences and associated metadata generated for this study are published in GISAID’s EpiCoV database. To view the contributors of each individual sequence with details such as accession number, Virus name, Collection date, Originating Lab and Submitting Lab and the list of Authors, visit 10.55876/gis8.230613ux.

## Supplementary Materials

The following supporting information can be downloaded at: https://www.mdpi.com/article/10.3390/v15061382/s1, Table S1: Frequency of cases classified as VOC/VOI by epidemiological week (EW) in Argentina; Tree S1: Maximum-likelihood phylogenetic tree of SARS-CoV-2 whole-genome sequences of Lambda (lineage C.37).
