# Supplementary material for: The Lambda Variant in Argentina: Analyzing the Evolution and Spread of SARS-CoV-2 Lineage C.37
Source: Viruses. 2023 Jun 16;15(6):1382. doi: 10.3390/v15061382 (PMC10305049; doi:10.3390/v15061382)
Supplement: Supplementary file 1 [file viruses-15-01382-s001.zip › viruses-2421970-supplementary.pdf]

Table S1. Frequency of cases classified as VOC/VOI by epidemiological week (EW) in Argentina[1].

EW	Alpha			Gamma			Lambda			Delta			Omicron BA.1			Omicron BA.2			Omicron BA.4/BA.5			Mu			non VOC/VOI		
	Freq (%)	95% CI[2]		Freq (%)	95% CI[2]		Freq (%)	95% CI[2]		Freq (%)	95% CI[2]		Freq (%)	95% CI[2]		Freq (%)	95% CI[2]		Freq (%)	95% CI[2]		Freq (%)	95% CI[2]		Freq (%)	95% CI[2]	
**until 53/2020**																									**100.00**	100.00	99.07
**1-2/2021**																									**100.00**	100.00	98.23
**3-4/2021**							**2.00**	7.00	0.36																**98.00**	99.64	93.00
**5-6/2021**	**1.61**	5.69	0.29	**1.61**	5.69	0.29	**1.61**	5.69	0.29																**95.16**	97.76	89.84
**7-8/2021**				**1.40**	4.96	0.25	**2.10**	5.99	0.57																**96.50**	98.50	92.08
**9-10/2021**	**6.61**	10.34	4.17	**4.67**	7.98	2.69	**8.56**	12.62	5.72	**0.39**	2.17	0.02													**79.77**	84.22	74.43
**11-12/2021**	**15.51**	20.02	11.87	**10.23**	14.16	7.30	**12.87**	17.11	9.56																**61.39**	66.69	55.79
**13-14/2021**	**8.97**	12.33	6.46	**29.89**	34.76	25.44	**27.99**	32.79	23.65																**33.15**	38.11	28.54
**15-16/2021**	**16.78**	20.58	13.56	**37.47**	42.11	33.05	**28.28**	32.68	24.25																**17.47**	21.32	14.19
**17-18/2021**	**8.89**	12.89	6.05	**45.56**	51.52	39.72	**31.11**	36.87	25.89													**0.37**	2.07	0.02	**14.07**	18.73	10.43
**19-20/2021**	**11.47**	15.09	8.63	**57.87**	62.76	52.81	**23.47**	28.01	19.46																**7.20**	10.27	5.00
**21-22/2021**	**8.63**	11.92	6.18	**62.53**	67.31	57.50	**23.99**	28.59	19.92																**4.85**	7.54	3.09
**23-24/2021**	**7.34**	10.69	4.98	**68.50**	73.30	63.28	**22.32**	27.14	18.15																**1.83**	3.94	0.84
**25-26/2021**	**7.42**	10.89	4.99	**64.84**	69.94	59.37	**26.45**	31.63	21.85																**1.29**	3.27	0.50
**27-28/2021**	**5.17**	8.12	3.25	**67.48**	72.31	62.24	**25.53**	30.51	21.12	**0.30**	1.70	0.02										**0.30**	1.70	0.02	**1.22**	3.08	0.47
**29-30/2021**	**5.40**	8.71	3.30	**66.55**	71.83	60.81	**24.46**	29.84	19.78	**1.44**	3.64	0.56										**0.72**	2.58	0.13	**1.44**	3.64	0.56
**31-32/2021**	**2.01**	4.08	0.97	**70.49**	75.03	65.50	**23.21**	27.92	19.09	**2.87**	5.19	1.56										**0.29**	1.60	0.01	**1.15**	2.91	0.45
**33-34/2021**	**3.17**	5.91	1.68	**67.25**	72.45	61.60	**22.89**	28.12	18.38	**5.63**	8.95	3.50										**0.35**	1.97	0.02	**0.70**	2.53	0.13
**35-36/2021**	**2.63**	5.33	1.28	**61.28**	66.93	55.30	**21.80**	27.15	17.26	**13.53**	18.17	9.94										**0.75**	2.70	0.13			
**37-38/2021**	**1.23**	4.39	0.22	**49.38**	57.01	41.79	**17.28**	23.85	12.24	**29.63**	37.07	23.14										**1.23**	4.39	0.22	**1.23**	4.39	0.22
**39-40/2021**	**0.68**	3.75	0.03	**33.33**	41.29	26.22	**16.33**	23.14	11.22	**48.98**	56.98	41.03										**0.68**	3.75	0.03			
**41-42/2021**	**3.67**	6.83	1.94	**17.14**	22.36	12.94	**17.14**	22.36	12.94	**61.63**	67.50	55.41													**0.41**	2.28	0.02
**43-44/2021**	**2.02**	4.65	0.87	**6.48**	10.26	4.03	**10.53**	14.98	7.29	**78.54**	83.20	73.01										**1.62**	4.09	0.63	**0.81**	2.90	0.14
**45-46/2021**	**0.33**	1.85	0.02	**3.31**	5.99	1.81	**12.91**	17.17	9.59	**82.78**	86.62	78.12										**0.66**	2.38	0.12			
**47-48/2021**							**3.35**	6.46	1.71	**96.65**	98.29	93.54															
**49-50/2021**				**0.24**	1.37	0.01	**0.98**	2.49	0.38	**70.42**	74.63	65.82	**27.63**	32.15	23.52										**0.73**	2.13	0.20
**51-52/2021**										**33.33**	37.50	29.41	**66.67**	70.59	62.50												
**1-2/2022**										**6.64**	9.42	4.63	**93.36**	95.37	90.58												
**3-4/2022**							**0.21**	1.18	0.01	**0.63**	1.83	0.17	**99.16**	99.67	97.86												
**5-6/2022**													**99.52**	99.98	97.33	**0.48**	2.67	0.02									
**7-8/2022**													**95.92**	98.12	91.38	**4.08**	8.62	1.88									
**9-10/2022**													**93.26**	96.87	86.06	**6.74**	13.94	3.13									
**11-12/2022**													**80.00**	87.92	68.73	**20.00**	31.27	12.08									
**13-14/2022**													**55.71**	66.75	44.08	**44.29**	55.92	33.25									
**15-16/2022**							**1.59**	8.46	0.08				**34.92**	47.25	24.33	**60.32**	71.47	47.98	**3.17**	10.86	0.56						
**17/2022**													**16.13**	32.63	7.09	**83.87**	92.91	67.37									

[1]Only the cases that were randomly collected and did not present travel history or close contact with travelers are included; in cases with a known epidemiological link between samples, only one was included to estimate frequencies.

[2]The 95% confidence intervals (95% CI) were estimated with the Wilson / Brown method, implemented in the Graph Pad Prism v.9.2 program (San Diego, California, United States, www.graphpad.com).
